# Synchronously in vivo real‐time monitoring bacterial load and temperature with evaluating immune response to decipher bacterial infection

**DOI:** 10.1002/btm2.10656

**Published:** 2024-03-12

**Authors:** Huaixuan Sheng, Huizhu Li, Shunyao Li, Chengxuan Yu, Yueming Wang, Haichen Hu, Lu Fang, Fuchun Chen, Yanyan Lu, Xiaogang Xu, Xing Yang, Shiyi Chen, Yuefeng Hao, Yunxia Li, Sijia Feng, Jun Chen

**Affiliations:** ^1^ Sports Medicine Institute of Fudan University, Department of Sports Medicine, Huashan Hospital Fudan University Shanghai China; ^2^ Department of Anatomy and Physiology School of Medicine, Shanghai Jiao Tong University Shanghai China; ^3^ University of Chinese Academy of Sciences Beijing China; ^4^ Key Laboratory of Infrared System Detection and Imaging Technology, Shanghai Institute of Technical Physics, Chinese Academy of Sciences Shanghai China; ^5^ Institute of Antibiotics, Huashan Hospital, Key Laboratory of Clinical Pharmacology of Antibiotics, National Health Commission, Fudan University Shanghai China; ^6^ Department of Orthopedics Affiliated Suzhou Hospital of Nanjing Medical University Suzhou China

**Keywords:** bacterial infection, immune response, in vivo, NIR‐II fluorescence imaging, temperature

## Abstract

Determining the precise course of bacterial infection requires abundant in vivo real‐time data. Synchronous monitoring of the bacterial load, temperature, and immune response can satisfy the shortage of real‐time in vivo data. Here, we conducted a study in the joint‐infected mouse model to synchronously monitor the bacterial load, temperature, and immune response using the second near‐infrared (NIR‐II) fluorescence imaging, infrared thermography, and immune response analysis for 2 weeks. *Staphylococcus aureus* (*S. aureus*) was proved successfully labeled with glucose‐conjugated quantum dots in vitro and in subcutaneous‐infected model. The bacterial load indicated by NIR‐II fluorescence imaging underwent a sharp drop at 1 day postinfection. At the same time, the temperature gap detected through infrared thermography synchronously brought by infection reached lowest value. Meanwhile, the flow cytometry analysis demonstrated that immune response including macrophage, neutrophil, B lymphocyte, and T lymphocyte increased to the peak at 1 day postinfection. Moreover, both M1 macrophage and M2 macrophage in the blood have an obvious change at ~ 1 day postinfection, and the change was opposite. In summary, this study not only obtained real‐time and long‐time in vivo data on the bacterial load, temperature gap, and immune response in the mice model of *S. aureus* infection, but also found that 1 day postinfection was the key time point during immune response against *S. aureus* infection. Our study will contribute to synchronously and precisely studying the complicated complex dynamic relationship after bacterial infection at the animal level.

**Translational Impact Statement**

Bacterial infection was a major and enduring threat to public health. Deciphering the bacterial infection from multiple dimensions synchronously will enrich more precise evidence to current knowledge. In this study, we innovatively monitored the bacterial infection from the bacterial load, temperature gap, and immune response of *Staphylococcus aureus* infection and found that 1 day postinfection was the key time point during immune response against *S. aureus* infection. These findings will lay the foundation for future interpretations of the mechanisms behind bacterial infection.

## 1 INTRODUCTION

Elucidating the precise course of bacterial infection is a topic of interest for developing anti‐infection treatments.^1^ Without unraveling the precise course of infection, we cannot utilize the narrow window for diagnosis or improve the efficacy of patient management. Therefore, it is essential to decipher the precise course of bacterial infection, which was a major and enduring threat to public health, with significant social and medical impact.^2^, ^3^, ^4^, ^5^, ^6^, ^7^, ^8^

Previous studies have revealed that three key factors including the bacterial load, temperature, and immune response affect each other during bacterial infection.^9^, ^10^ When bacterial infection occurs, the temperature of the infected organism increases, triggering a heat shock response in the body.^11^, ^12^ Bacteria secrete toxins that trigger inflammatory responses and activate immune cells.^13^ In addition, a typical proinflammatory response to infection has been shown to be closely linked to the elevation in temperature.^12^, ^14^ As there was limited evidence for mutual interactions based on asynchronous in vitro studies, a significant gap in knowledge existed due to the lack of synchronized in vivo analysis of the three factors, thus preventing further comprehension of the communication among these three factors after infection.

As emerging in vivo optical imaging technologies, both the second near‐infrared (NIR‐II, 1000–1700 nm) fluorescence imaging and infrared thermography have recently attracted broad attention.^15^, ^16^ Both imaging technologies have the advantages of noninvasiveness and excellent spatiotemporal resolution for biomedical applications, especially for in vivo studies.^17^, ^18^ In our previous studies, NIR‐II fluorescent lead sulfide quantum dots (QDs) with ribonuclease‐A (RNase‐A) protein coating were found to be a biocompatible imaging agent with excellent NIR‐II fluorescence characteristics.^19^, ^20^, ^21^, ^22^ They had the capacity to detect labeled bacteria, and the fluorescence signals could further efficiently detect and dynamically monitor subcutaneous bacterial infection, relying on their interactions with bacterial membranes.^23^ At the same time, with growing concern regarding the profound significance of temperature, infrared thermography has enabled various insights into phenomena relating to normal physiology and disease,^24^, ^25^, ^26^, ^27^ including bacterial infection and inflammation. Moreover, because infrared thermography provides real‐time information without harmful radiation or the addition of contrasting dyes, in vivo temperature data can be obtained and analyzed at a high level of biosafety.^24^ By combining these two cutting‐edge real‐time monitoring techniques, the fluorescence signal data of the targeted substances and temperature data of specific regions at the same time point in the same experimental animal can be continuously obtained, achieving multidimensional monitoring of real‐time data in living organisms. Compared with in vitro experiments, this combined strategy can provide far more authentic data for bacterial infection to gain insight into real‐time processes. Therefore, NIR‐II imaging and infrared thermography have the potential to facilitate the exploration of the relationship between the bacterial load, temperature, and immune response.

**FIGURE 1:**
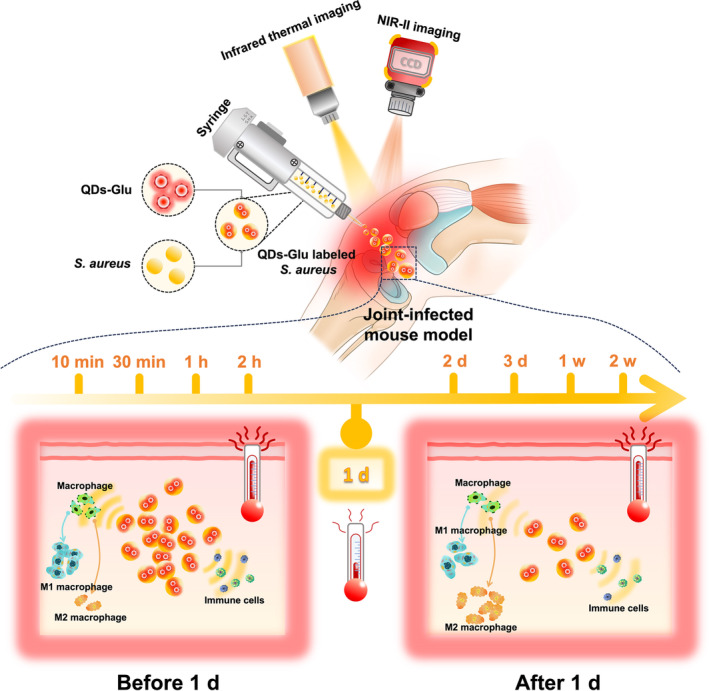
Schematic representation of utilization of the second near‐infrared (NIR‐II) fluorescence imaging, infrared thermography and immune response analysis to identify the key time point of bacterial infection in a joint‐infected mouse model. QDs‐Glu, glucose‐conjugated quantum dots.

Here, we presented a NIR‐II fluorescence imaging strategy based on glucose‐conjugated quantum dots (QDs‐Glu), which not only demonstrated excellent NIR‐II fluorescence properties as previously shown,^22^ but also facilitated the monitoring of bacterial infection in vivo in real‐time (Figure 1). First, the QDs‐Glu were prepared, which acted as an effective target to label *Staphylococcus aureus* (*S. aureus*) cultured in vitro and track it in vivo. Subsequently, the photoluminescence (PL) intensity ratio of QDs‐Glu‐*S. aureus* (*S. aureus* labeled by QDs‐Glu) and the temperature of three specific areas over time were recorded to analyze the dynamic changes in bacterial load and temperature variation in vivo in a joint‐infected mouse model. Flow cytometry analysis was performed longitudinally to evaluate the corresponding immune responses to bacterial infection. This study aimed to decipher the triadic relationship between bacterial infection under the guidance of NIR‐II fluorescence imaging, infrared thermography and immune response analysis, thus laying the foundation for future interpretations of the mechanisms behind the triadic relationships.

## 2 MATERIALS AND METHODS

### 2.1 Reagents and materials

All reagents were purchased as products and used without further modification. Working solutions were prepared using deionized water generated using an ELGA Purelab Classic UVF system (ELGA LabWater, Woodridge, IL, USA). Bovine pancreatic RNase‐A (MW: 13.7 kDa, >70 U/mg), lead acetate trihydrate (Pb(OAc)_2_·3H_2_O, ≥99.9%), sodium sulfide nonahydrate (Na_2_S·9H_2_O, ≥98.0%), sodium hydroxide (NaOH, ≥98.0%), and N‐hydroxysuccinimide (NHS) were purchased from Sigma‐Aldrich (St Louis, MO, USA). 1‐Ethyl‐3‐(3‐dimethylaminopropyl) carbon diamine hydrochloride (EDC) was purchased from Thermo Fisher Scientific (Waltham, MA, USA). Sterile 1× phosphate‐buffered saline (PBS) with a pH of 7.4 was used as mentioned.

### 2.2 Bacteria

*S. aureus* (ATCC 29213) and *Staphylococcus epidermidis* (*S. epidermidis*, clinical isolates) were strains utilized and were obtained from the Institute of Antibiotics, Huashan Hospital, Fudan University, Shanghai, China.

### 2.3 Animals

Male BALB/c mice (8 weeks old, 24–28 g) were purchased from Shanghai Jie Si Jie Laboratory Animal Co., Ltd. (Shanghai, China). All animal experiments were performed in strict accordance with the Chinese Council for Animal Care guidelines. This work received the approval of ethics by the Ethics Committee of Fudan University (2022JS‐486).

### 2.4 Instrumentation

A microwave reactor (Discover; CEM Corporation, Matthews, NC, USA) with a cooling system was used for the microwave synthesis of QDs. Freshly synthesized QDs were purified by ultracentrifugation in ultracentrifugal filter tubes (Amicon, Darmstadt, Germany). Excitation shadowless illumination was provided by a fiber‐coupled 808 nm laser. The excitation light power was 100 mW/cm^2^. In total, 1300 nm bandpass filter and 1100 nm long‐pass filter were used respectively to filter the emitted light before it reached the camera. The emitted light was collimated using a 50 mm focal length NIR‐II lens (Goldeye G‐130 TEC1; Allied Vision, Shanghai, China). Fluorescence was detected using a liquid‐nitrogen‐cooled InGaAs camera (NIR vana 640; Teledyne Princeton Instruments, Trenton, NJ, USA) located immediately above the working stage, irradiating the subject vertically.

### 2.5 Synthesis of QDs

The synthesis protocol of QDs was based on our previously published protocol.^20^ First, 500 μL of Pb(OAc)_2_ solution (10 mM) and RNase‐A solution (50 mg/mL) were mixed to prepare the RNase‐A/Pb^2+^ solution, which was added to a reaction tube. Then, 50 μL of NaOH solution (1 M) was used to adjust the pH of the system to 9–11 with stirring for 5 min. After that, 50 μL of freshly prepared Na_2_S solution (10 mM) was added. A stir bar was placed in the mixture to ensure even heating before the reaction tube was inserted into the microwave reactor. When the QDs were freshly prepared, the solution turned dark brown. Finally, the solution was ultrafiltered for purification and rinsed with PBS to remove excess reagents. The as‐prepared QDs were stored at 4°C in the dark.

### 2.6 Bacterial culture

*S. aureus* and *S*. *epidermidis* strains were first cultured overnight in tryptic soy broth (TSB) medium at 37°C under 5% CO_2_ in an incubator shaker (150 rpm). A single colony was then inoculated into fresh TSB medium (4 mL) and cultured to an optical density of 0.5–0.6 at 600 nm. The *S. aureus* suspension was then centrifuged at 2000 rpm for 15 min at 25°C. Bacterial sediments were collected after rinsing twice with PBS and were resuspended in 5 mL PBS. Finally, the bacterial suspension was diluted to different concentrations for colony‐forming unit (CFU) calculations.

### 2.7 *S. aureus* labeling by QDs‐Glu

The pH of the QDs solution was adjusted to 5–6 using NaOH and HCl, after which, EDC and NHS were added to QDs solution, and the mixture was vibrated at room temperature for 40 min. Next, glucose (G6172, Macklin, Shanghai, China) was added to the mixture according to previous protocol.^28^ After an overnight reaction at 4°C, the mixture was centrifuged using a ultrafiltration tube to remove unbound impurities and QDs‐Glu was prepared. *S. aureus* was then co‐incubated with QDs‐Glu (*S. aureus*: QDs‐Glu fixed at 1:2) overnight at 37°C according to previous protocol.^29^

### 2.8 Preparation of experimental animal models

The mice were anesthetized using isoflurane at a flow rate of 1.5 L/min, while oxygen was simultaneously administered at a flow rate of 0.2 L/min throughout the injections and in vivo imaging. The infected mice were intra‐articularly injected with 60 μL of QDs‐Glu‐*S. aureus* at a bacterial concentration of 10^8^ CFU/mL in the left knee. The concentration was set based on previous protocols.^23^, ^29^

### 2.9 In vivo observation with NIR‐II imaging

The mice were housed in cages under identical conditions. From the start of the observation, NIR‐II fluorescence images were taken from the same mouse over a time course (10, 30 min, 1, 2 h, 1, 2, 3 days, 1, and 2 weeks postinfection). The PL intensity ratio was measured based on the knee joint area injected with labeled *S. aureus*. ImageJ (version 1.51; National Institutes of Health, Bethesda, MD, USA) was used to standardize all NIR‐II fluorescence images and analyze PL intensity data. To avoid background interference, the PL intensity ratio, calculated as the PL intensity at the injected site/PL intensity of the background, and the maximum PL intensity ratio, calculated as the maximum PL intensity at the injected site/ PL intensity of the background were also calculated according to previous protocols.^21^, ^29^, ^30^

### 2.10 In vivo observation with thermal imaging

For thermal imaging, the skin temperature of the infected knee was recorded using a TAS system (InfiRay, Shandong, CHINA). Thermal images were taken of the mice over a time course similar to that of NIR‐II imaging (10, 30 min, 1, 2 h,1, 2, 3 days, 1, and 2 weeks postinfection). To avoid fluctuations in temperature caused by the different statuses of the mice, the temperature gap calculated as T_IH_ = Temperature_infected knee_ – Temperature_healthy knee_, T_AI_ = Temperature_abdomen_ – Temperature_infected knee_ was recorded.^31^ Either T_IH_ getting higher or T_AI_ getting lower after injection indicated that the temperature of the infected knee was relatively higher than before being infected.

### 2.11 Flow cytometry analysis

One hundred microliters of freshly collected blood from the angular vein of each mouse were mixed with 2 mL of ACK Lysis Buffer (Thermo Fisher, Waltham, MA, USA) for 10 min. The sample was then centrifuged at 1500 rpm for 5 min and rinsed with 1 mL of PBS. After fluorescent staining with antibodies for 30 min at 4°C, the sample was rinsed again with 1 mL of PBS and resuspended in 300 μL of PBS. The percentage of immune cells (macrophage, M1 macrophage, M2 macrophage, neutrophil, B lymphocyte, CD4^+^T lymphocyte, CD8^+^T lymphocyte) in the blood was calculated and analyzed.

### 2.12 Immunofluorescence staining

After implantation for 1 week, the tissues were carefully dissected, fixed in paraformaldehyde for 2 weeks, and decalcified in 10% EDTA for 8 weeks. Tissue sections (~5 μm) were cut after embedding in paraffin. Immunofluorescence staining was conducted to evaluate the expression of TNF‐α, IL‐1β, and Arg‐1 in tissues. The positive cell density was analyzed and calculated, reflecting the degree of positivity of M1 macrophage and M2 macrophage.

### 2.13 Blood culture

One hundred microliters of freshly collected blood from the angular vein of each mouse were inoculated on blood agar plates. Bacterial colony counting was performed after the samples were cultured overnight at 37°C under 5% CO_2_.

### 2.14 Histopathological analysis

Tissues from the left knee at different time points and major organs (2 weeks postinfection) of the mice were collected and stored in 4% paraformaldehyde for at least 24 h to prepare slides for histopathological analysis. After hematoxylin and eosin (H&E) and Gram staining, histopathological images were acquired using an optical microscope (Nikon Eclipse CI; Nikon, Tokyo, Japan). Images of the immunohistochemical (IHC) sections were captured using a tissue section digital scanner and imaging system, and the tissue measurement area was automatically read using the Seville image analysis system. The positive area and positive cells were analyzed and calculated, reflecting the degree of positivity of neutrophil stained with CD11b antibody.

### 2.15 Reverse transcription‐polymerase chain reaction analysis of heat shock protein 60, heat shock protein 70, and heat shock protein 90

RNA was extracted using the TRIZOL method (Invitrogen, Carlsbad, CA, USA), and complementary DNA was synthesized from 1 μg of total RNA using iSCRIPT (BIO‐RAD, Hercules, CA, USA) reverse transcriptase. Reverse transcription‐polymerase chain reaction (RT‐PCR) analysis of HSP60, HSP70, and HSP90 was performed using primer sequences and conditions reported previously.^32^, ^33^, ^34^ The products were electrophoresed on 2% agarose gels containing 0.5 μg/mL ethidium bromide. mRNA expression levels were normalized to *GAPDH* expression levels using the 2^△Ct^ method. The mean expression level of the heat shock proteins (HSPs) mRNA (2^−△Ct^) at 1 day postinfection was selected as the baseline.

### 2.16 Statistical analysis

Comparisons were performed and analyzed using GraphPad Prism 8.0 (GraphPad, San Diego, CA, USA). A Student's *t*‐test was used to compare the mean values before 1 day postinfection with the mean values after 1 day postinfection. The mean value was calculated using data from all time points (three samples or more at each time point) before or after 1 day postinjection. A *p* < 0.05 indicated statistical significance. Details of different *p*‐values, with associated asterisks, belong in the appropriate figure legends.

## 3 RESULTS AND DISCUSSION

### 3.1 Evaluating the labeling capability of QDs‐Glu for bacteria in vitro and in vivo

**FIGURE 2:**
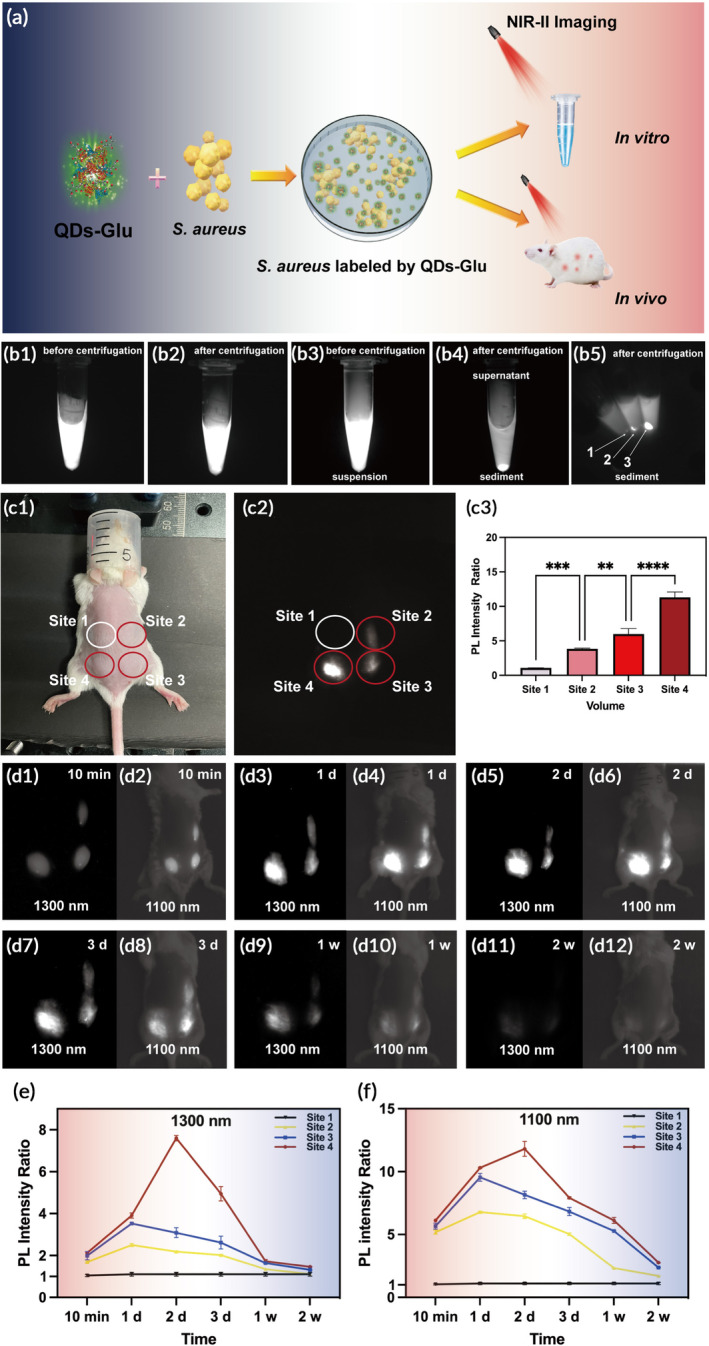
(a) Schematic of testifying the labeling capability of glucose‐conjugated quantum dots (QDs‐Glu) to *S. aureus* in vitro and in vivo. The second near‐infrared (NIR‐II) fluorescence images of (b1,b2) QDs‐Glu, (b3,b4) QDs‐Glu‐*S. aureus*, and (b5) QDs‐Glu‐*S. aureus* at different concentrations (1: 10^4^ CFU/mL, 2: 10^6^ CFU/mL, 3: 10^8^ CFU/mL). (c1) Bright field photographs of subcutaneous‐infected mouse model. (c2) NIR‐II fluorescence images of subcutaneous‐infected mouse model at 1 day postinfection (Site 1: 10 μL PBS; Site 2: 10 μL QDs‐Glu‐*S. aureus*; Site 3: 25 μL QDs‐Glu‐*S. aureus*; Site 4: 50 μL QDs‐Glu‐*S. aureus*). (c3) Graphs of PL intensity ratio analyzed from (c2). (d1–d12) NIR‐II fluorescence images of the same mouse during postinfection with 1100 nm and 1300 nm filter. (e,f) Graphs of photoluminescence (PL) intensity ratio analyzed from (d1–d12). **p < 0.01,***p < 0.001, ****p < 0.0001.

A schematic demonstrated the synthesis of QDs‐Glu and verification of its labeling capacity of *S. aureus* was shown in Figure 2a. First, 200 ms was chosen as the exposure time for observation (Figure S1a1–a7). Second, to evaluate the labeling capability of QDs‐Glu in vitro, NIR‐II fluorescence images of QDs‐Glu (Figure 2b1,b2), QDs‐Glu‐*S. aureus* (Figure 2b3,b4) were obtained. It was shown that, in contrast to QDs‐Glu, which showed no sediments after centrifugation (Figure 2b1,b2), strong fluorescence signals were detected in the bacterial sediments of QDs‐Glu‐*S. aureus* after centrifugation (Figure 2b3,b4), indicating successful labeling of the bacteria by QDs‐Glu. Furthermore, QDs‐Glu were co‐cultured with three concentrations (10^4^, 10^6^, and 10^8^ CFU/mL) of *S. aureus*, and the PL intensity ratio increased with higher *S. aureus* concentration, suggesting that a higher PL intensity ratio indicated a higher concentration of bacteria (Figure 2b5). Furthermore, the stability of QD‐Glu‐*S. aureus* was tested at 37°C by co‐culturing QDs‐Glu with *S. aureus* for 24 h. The fluorescence signal remained stable from 3 h to 24 h, which supported the suitability of QDs‐Glu‐*S. aureus* for use in vivo (Figure S1b,c). Thus, QDs‐Glu could label various concentrations of *S. aureus* and had good stability at different temperatures.

In addition, to evaluate the in vivo labeling capability of QDs‐Glu to bacteria, they were injected subcutaneously into the back of mice. First, 10 μL PBS (Site 1) and three volumes of QDs‐Glu‐*S. aureus* (Site 2–10 μL, Site 3–25 μL, and Site 4–50 μL) were injected subcutaneously into four sites on the back (Figure 2c1,c2). The PL intensity ratio differed significantly among four sites (*p* < 0.01, Figure 2c3). Further, from 10 min to 2 weeks postinjection, the PL intensity ratio of Site 2 and Site 3 peaked at 1 day and then decreased, whereas Site 4 peaked at 2 days and then decreased and Site 1 showed no fluorescence at exposure time of 100 ms (Figure 2d–f). These results demonstrated the ability of QDs‐Glu to display differences when various volumes of bacteria were labeled in vitro and in vivo.

Besides, the labeling capability of the QDs‐Glu for bacteria in the knee joint was tested. First, QDs‐Glu were injected into the knee joint, and the results showed that the PL intensity ratio kept decreasing after injection (Figure S2). Besides, QDs‐Glu were also co‐cultured with 10^6^ CFU/mL (Figure S3a1–a4) and 10^8^ CFU/mL (Figure S3b1–b4) of *S. epidermidis* and then injected into the knee joint. The PL intensity ratio of the knee joint increased from 10 min to 1 h, peaked at 1 h, and then decreased at both concentrations tested (Figure S3c–d). In addition, higher concentration of bacteria in infected knee joint demonstrated higher PL intensity ratio (Figure S3e). These results indicated that QDs‐Glu could label bacteria, especially *Staphylococci* both subcutaneously and in the knee joint in vivo.

In summary, both high sensitivity and good stability of QD‐Glu were achieved in vitro and in vivo for NIR‐II imaging, which was promising for the visualization and monitoring of the long‐term translocation of *S. aureus* for biomedical applications in vivo.

### 3.2 Monitoring the bacterial load based on NIR‐II fluorescence imaging in vivo

**FIGURE 3:**
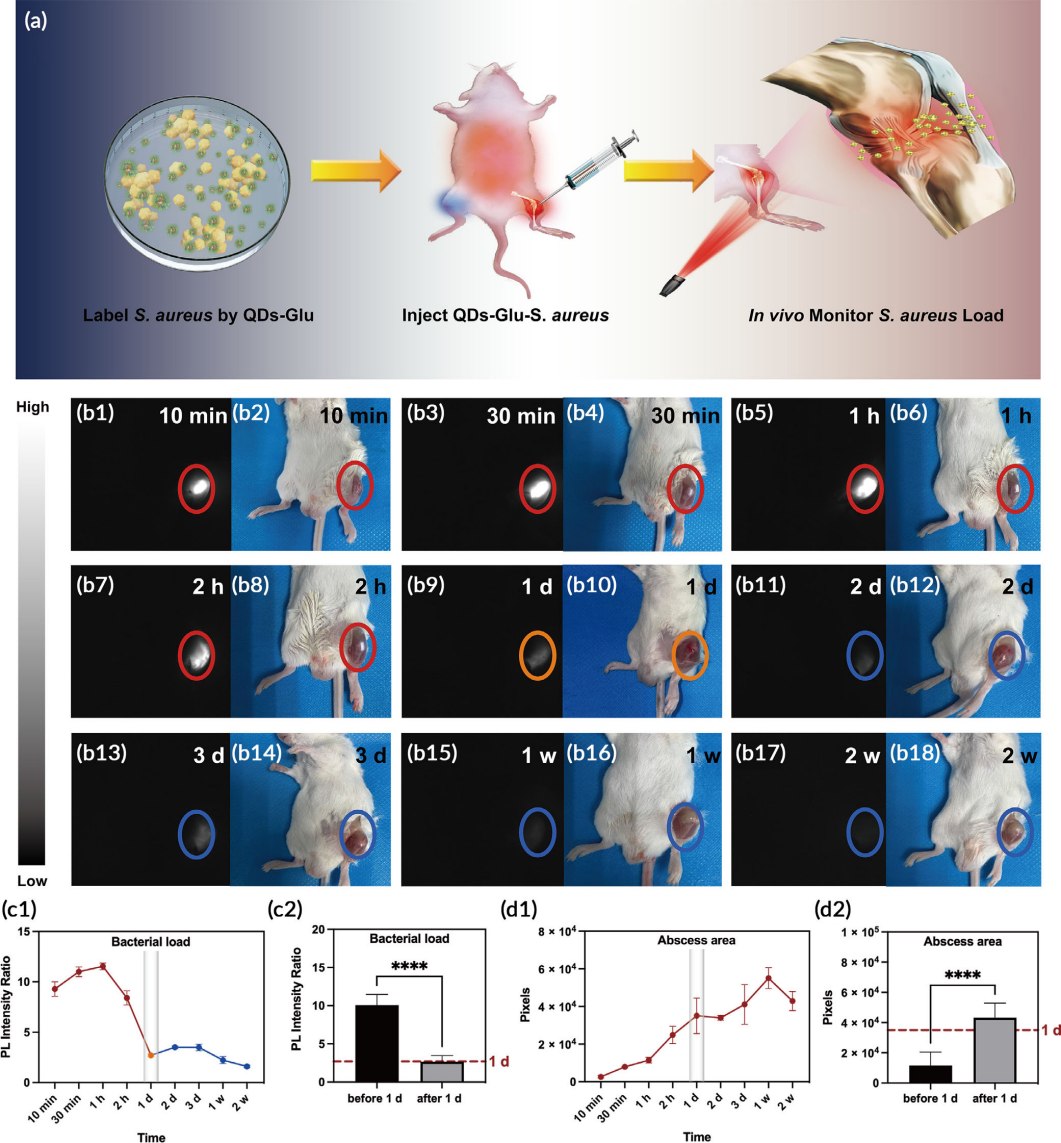
(a) Schematic to represent the in vivo the second near‐infrared (NIR‐II) fluorescence imaging process. (b1–b18) NIR‐II fluorescence images and bright field photographs of the same mouse postinfection. (c1–d2) Graphs of bacterial load and abscess area analyzed from (b1–b18). Red dot line showed the 1 day level in the graphs (c2,d2). ****p < 0.0001.

With the evidence that QDs‐Glu possessed bacteria‐labeling ability both in vitro and in vivo, real‐time monitoring of the load of *S. aureus* in the knee joint was conducted. A joint‐infected mouse model induced by *S. aureus* was established, as *S. aureus* is one of the major bacterial strains causing joint infections^35^ (Figure 3a). NIR‐II fluorescence images and bright field photographs of the mouse joint at different time points postinfection were obtained with the corresponding PL intensity ratio and abscess areas were calculated (Figure 3b1–b18). An increase in PL intensity ratio was first detected from 10 min to 1 h and then decreased until 1 day, followed by a small increase that peaked at 3 days and then decreased thereafter (Figure 3c1). The NIR‐II fluorescence images captured at exposure times of 30 ms (Figure S4a1–a9), 50 ms (Figure S4b1–b9), 100 ms (Figure S4c1–c9), and 300 ms (Figure S4d1–d9) displayed the same trend. Furthermore, quantitative analysis based on the qualitative observations showed that the PL intensity ratio before 1 day postinfection was significantly higher than that after 1 day postinfection (*p* < 0.0001) at all exposure times (Figures 3c2 and S4a10,a11,b10,b11,c10,c11,d10,d11). The maximum PL intensity ratio showed the similar trend as above (Figure S5a) and the fluorescence area kept decreasing during infection (Figure S5b). These results suggested that *S. aureus* was labeled with QDs‐Glu at the infected joint in vivo instead of being diluted and rapidly metabolized. In addition, a joint‐infected mouse model caused by *S. epidermidis* was established, it was also observed that the bacterial load decreased sharply at 1 day postinfection (*p* < 0.0001, Figure S6). Furthermore, the infected knee of the mouse presented swelling at 1–3 days postinfection and festered thereafter (Figure 3d1) and the abscess area was significantly lower before 1 day than that after 1 day postinfection (*p* < 0.0001, Figure 3d2). In summary, the results indicated that joint infection in mice could be visualized using QDs‐Glu‐*S. aureus* based on NIR‐II fluorescence imaging in vivo, and 1 day postinfection was found to be the key time point, as the load of *S. aureus* plummeted at ~1 day postinfection.

To investigate the spread of infection, blood cultures were conducted based on the blood collected from joint‐infected mouse model at a series of time points postinfection (Figure S7a1–a9). *S. aureus* colonies increased from 10 min to 2 days postinfection and then decreased (Figure S7b). Compared with the result that PL intensity ratio peaked at 1 h postinfection, the delay of the peak might be due to the fact that bacteria required time to escape from intra‐articular space to vessels.^36^

### 3.3 Monitoring the temperature during infection using infrared thermography in vivo

**FIGURE 4:**
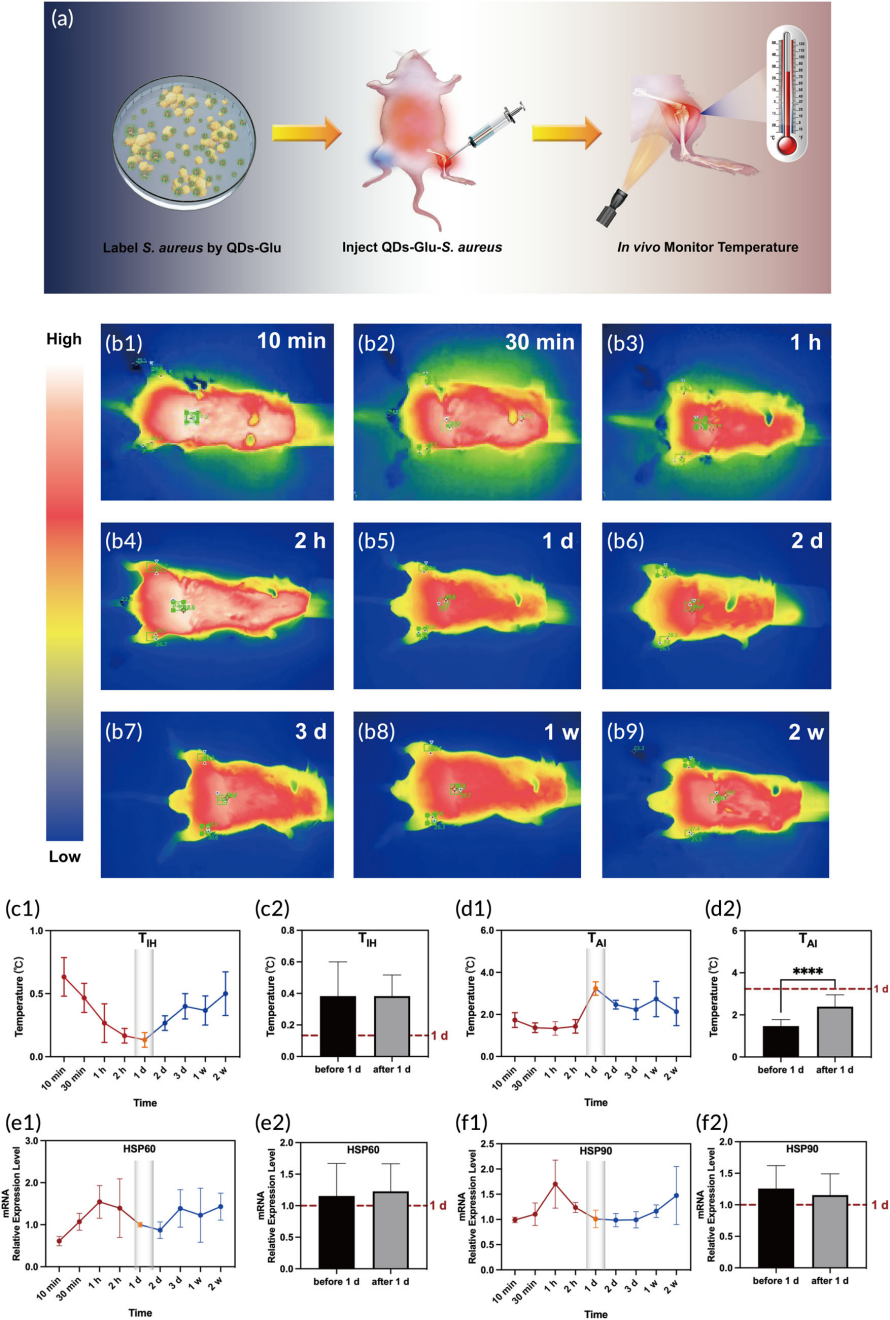
(a) Schematic to represent the in vivo infrared thermography process. (b1–b9) infrared thermal images of the same mouse postinfection. (c1–d2) Graphs of T_IH_ and T_AI_ analyzed from (b1–b9). Graphs of (e1,e2) HSP60 and (f1,f2) HSP90 mRNA expression level. Red dot line showed the 1 day level in the graphs (c2,d2,e2,f2). ****p < 0.0001.

Infrared thermography has been increasingly applied to monitor skin temperature in clinical settings to assist in the analysis and evaluation of inflammation and the immune response.^37^, ^38^, ^39^ In general, the temperature was higher where inflammation was more severe.^40^, ^41^ In the current study, the skin temperatures of the knees and abdomen were measured bilaterally (Figure 4a). Thermal infrared images were captured at different time points (Figure 4b1–b9). The temperature in the three areas continued to increase until 3 days postinfection (Figure S8a). Then, a decrease was observed until the observation ended at 2 weeks and the temperature at all times after infection was higher than that before infection (Figure S8a). Furthermore, based on the temperatures of the three areas, both T_IH_ (Figure 4c1) and T_AI_ (Figure 4d1) were calculated and analyzed to account interindividual differences. T_IH_ decreased until 1 day, followed by a constant increase, and T_AI_ increased until 1 day and then decreased, indicating the same trend. Additionally, based on the qualitative observation of the trend, quantitative analysis was conducted. The T_IH_ and T_AI_ before 1 day was compared with that after 1 day postinfection respectively. The T_IH_ was similar before and after 1 day postinfection (Figure 4c2). However, T_IH_ at 1 day postinfection was the lowest during bacterial infection. Besides, the T_AI_ was significantly lower before 1 day than after 1 day postinfection (*p* < 0.0001, Figure 4d2), and the T_AI_ at 1 day postinfection was the highest during bacterial infection. These results based on temperature monitoring demonstrated that 1 day postinfection also played the role of a turning point, which confirmed the importance of 1 day postinfection over the other time points.

The heat‐shock response is closely related to a higher body temperature caused by bacterial infection, and this is accompanied by high expression levels of HSPs.^11^ In this study, the expression patterns of HSP60, HSP70, and HSP90 were analyzed because of their vital roles in the response to bacterial infection.^42^ RT‐PCR results showed that the expression levels of HSP60 (Figure 4e1), HSP90 (Figure 4f1), and total HSP (HSP60 + HSP70 + HSP90) increased till 1 h postinfection and began to decrease (Figure S8b); however, the expression level of HSP70 (Figure S8c) remained stable throughout the observation period. The expression levels of HSP60 and HSP90 before 1 day were also compared with their levels after 1 day postinfection, but they were close (Figure 4e2,f2).

### 3.4 Analyzing the immune response to bacterial infection based on systemic and local analyses

**FIGURE 5:**
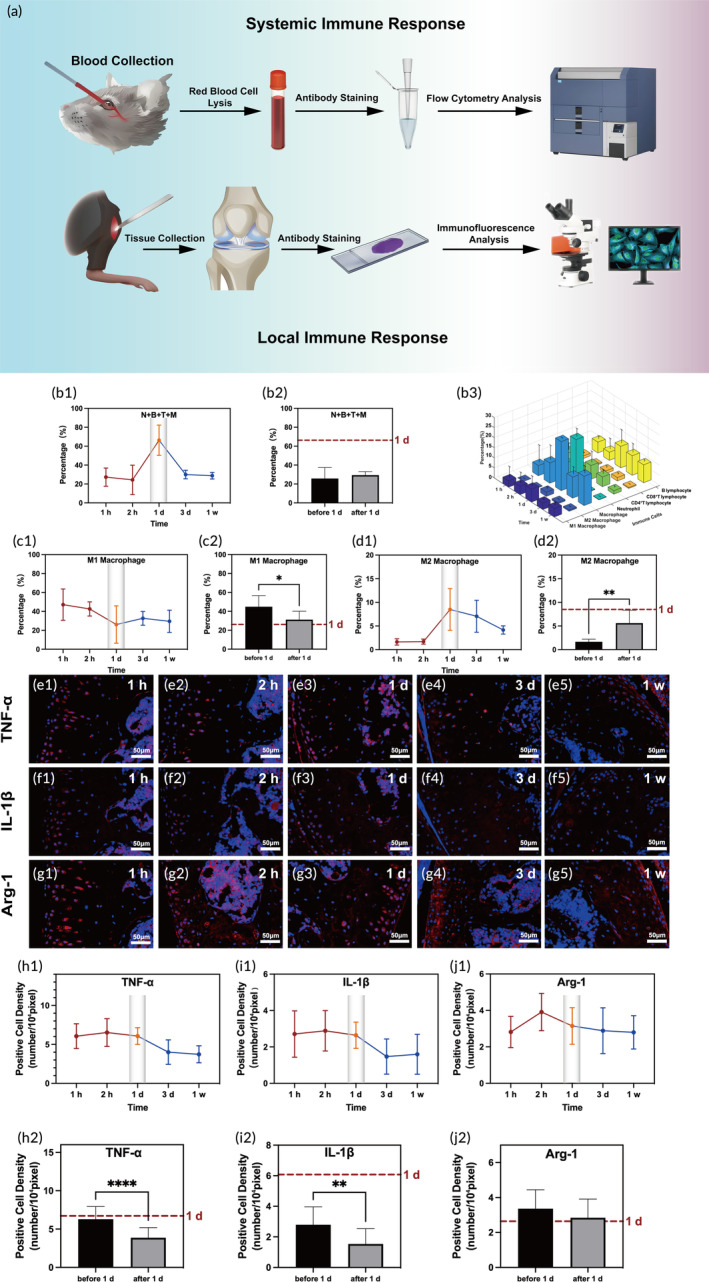
(a) Schematic of systemic and local immune response evaluation. Graphs of (b1,b2) N + B + T + M cells (N: neutrophil; B: B lymphocyte; T: T lymphocyte, M: macrophage), (b3) total immune cells (macrophage, neutrophil, B lymphocyte, and T lymphocyte), (c1,c2) M1 macrophage, (d1,d2) M2 macrophage based on flow cytometry analysis. Representative immunofluorescence images stained with antibody of (e1–e5) TNF‐α, (f1–f5) IL‐1β, and (g1–g5) Arg‐1 in the harvested infected knee joint, and (h1–j2) graphs of positive cell density based on (e–g). Red dot line showed the 1 day level in the graphs (b2,c2,d2,h2,i2,j2). *p < 0.05, **p < 0.01, ****p < 0.0001.

Flow cytometry and immunofluorescence analyses were performed to investigate immune response after bacterial infection (Figure 5a). Immune cells (neutrophil, B lymphocyte, T lymphocyte, and macrophage) in the blood of infected mice peaked at 1 day postinjection (Figure 5b1), and immune cells before 1 day were similar to those after 1 day postinfection (Figure 5b2,b3). Macrophage was further analyzed because of their vital role in the innate immune response to bacterial infection.^43^ M1 macrophage decreased continously until 1 day postinfection and kept stable (Figure 5c1), and M1 macrophage was significantly higher before 1 day than that after 1 day (*p* < 0.05, Figure 5c2). M2 macrophage peaked at 1 day post‐infection and decreased until 1 weeks and M2 macrophage before 1 day was significantly lower than that after 1 day (*p* < 0.01, Figure 5d1,d2). The results of immunofluorescence analyses based on the harvested infected knee showed TNF‐α (Figure 5e1–e5) and IL‐1β (Figure 5f1–f5) positive cells underwent a sudden drop at 3 days postinfection (Figure 5h1–i2). Moreover, the Arg‐1 (Figure 5g1–g5) positive cells peaked at 2 h and then began to decrease (Figure 5j1,j2) till 1 week postinfection. These above results indicated that in local, M1 macrophage dropped after 1 day post‐infection, whereas M2 macrophage increased from low level to its peak before 1 day post‐infection. Although the qualitative trends of macrophage analyzed by flow cytometry and immunofluorescence were similar, the peak were observed at different time point. Decrease of M1 macrophage in the blood was earlier than the observed time of that in local. At the same time, increase of M2 macrophage in the blood was later than that observed in the infected knee. This difference indicated that though systemic immune response was closely related to the local immune response, it was not to the timeline. As previous studies have shown, the opposite trend of M1 and M2 polarization after infection might indicate that a lasting bacterial infection in the joint may induce M2 polarization,^44^ which makes the tissue under localized infection anti‐inflammatory and fibrotic, leading to a chronic infection.^43^, ^45^, ^46^, ^47^ In addition, the migration of immune cells to the infection site requires time, which leads to a delay in M1 macrophage reduction of number in local.^48^, ^49^, ^50^ As for the locally advanced M2 polarization, it was suggested that tissue repair was initiated first in the joint, then leading to M2 polarization systemically.^51^, ^52^, ^53^

**FIGURE 6:**
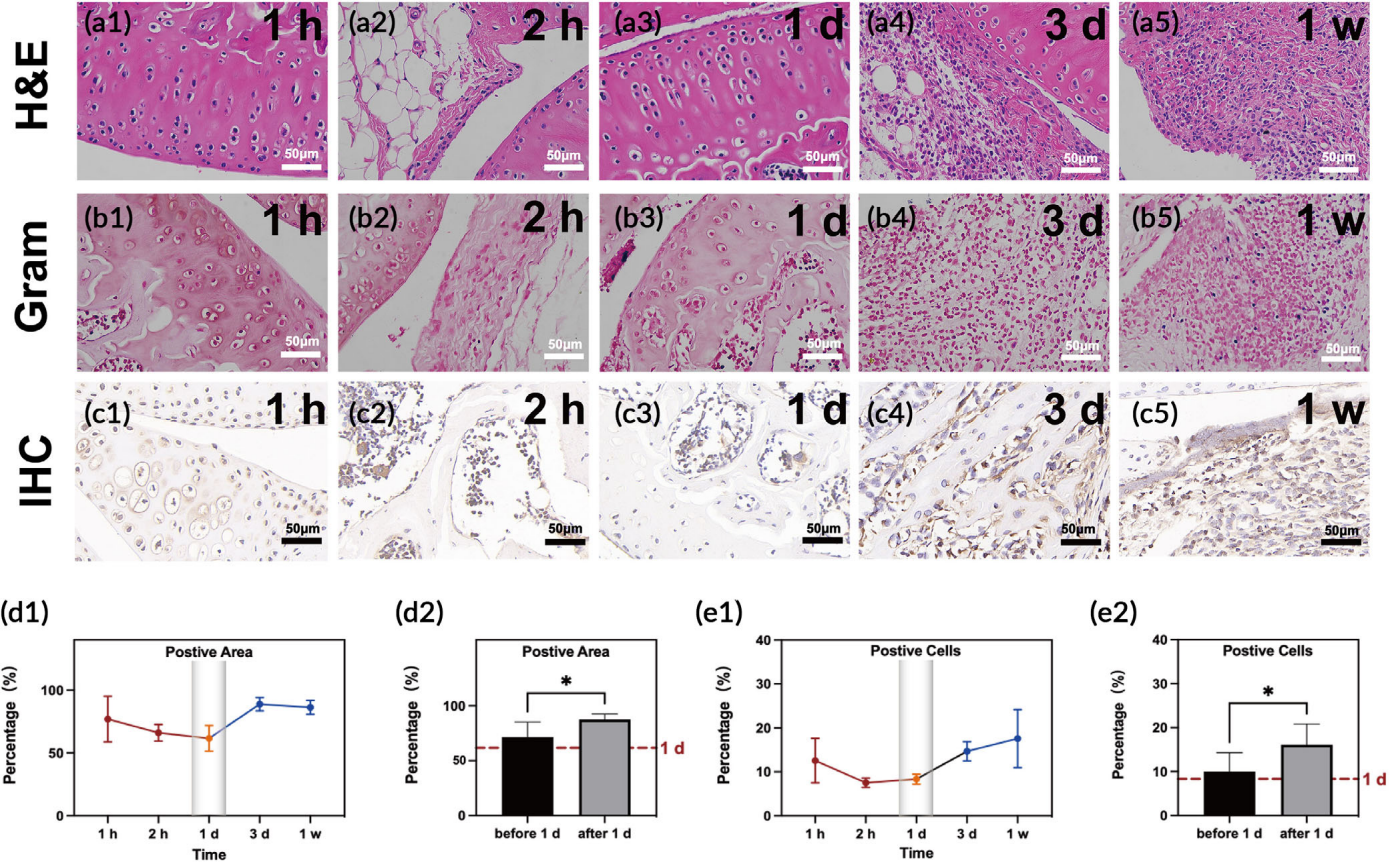
Representative micrographs of (a1–a5) hematoxylin and eosin staining (H&E), (b1–b5) Gram staining and (c1–c5) immunohistochemical (IHC) CD11b staining. (d1,d2,e1,e2) Graphs of positive area and positive cells analyzed from (c1–c5). Red dot line showed the 1 day level in the graphs (d2,e2). *p < 0.05.

Mice were also sacrificed at different time points postinfection in subsequent experiments, and the infected knee was harvested. First, H&E staining showed that there were sporadic inflammatory cells in the knee tissue of the infected mouse (Figure 6a1–a5). Second, *S. aureus* was found to increase based on Gram staining (Figure 6b1–b5). Third, IHC staining (Figure 6c1–c5) was conducted, and both qualitative and quantitative analyses of the CD11b positive area and CD11b positive cells based on IHC staining were measured and calculated to evaluate the distribution and quantity of neutrophil. The qualitative results suggested that neutrophil decreased, reached its lowest at 1 day postinfection, and then increased (*p* < 0.05, Figure 6d1–e2). In addition, quantitative analyses of the CD11b positive cells and CD11b positive area before and after 1 day postinfection showed that inflammation was strongly exacerbated after 1 day postinfection.

### 3.5 Validating the biosafety of the QDs‐Glu

Finally, the major organs of the mice that were intra‐articularly injected with QD‐Glu were collected 2 weeks postinjection to test the biosafety of QDs‐Glu‐based NIR‐II fluorescence imaging. Major organs, including the intestine, lung, liver, brain, spleen, kidney, stomach, and heart were collected. No obvious abnormalities were observed (Figure S9), ensuring the biosafety of QDs‐Glu.

### 3.6 Deciphering the triadic relationship between bacterial infection, temperature, and the immune response

**FIGURE 7:**
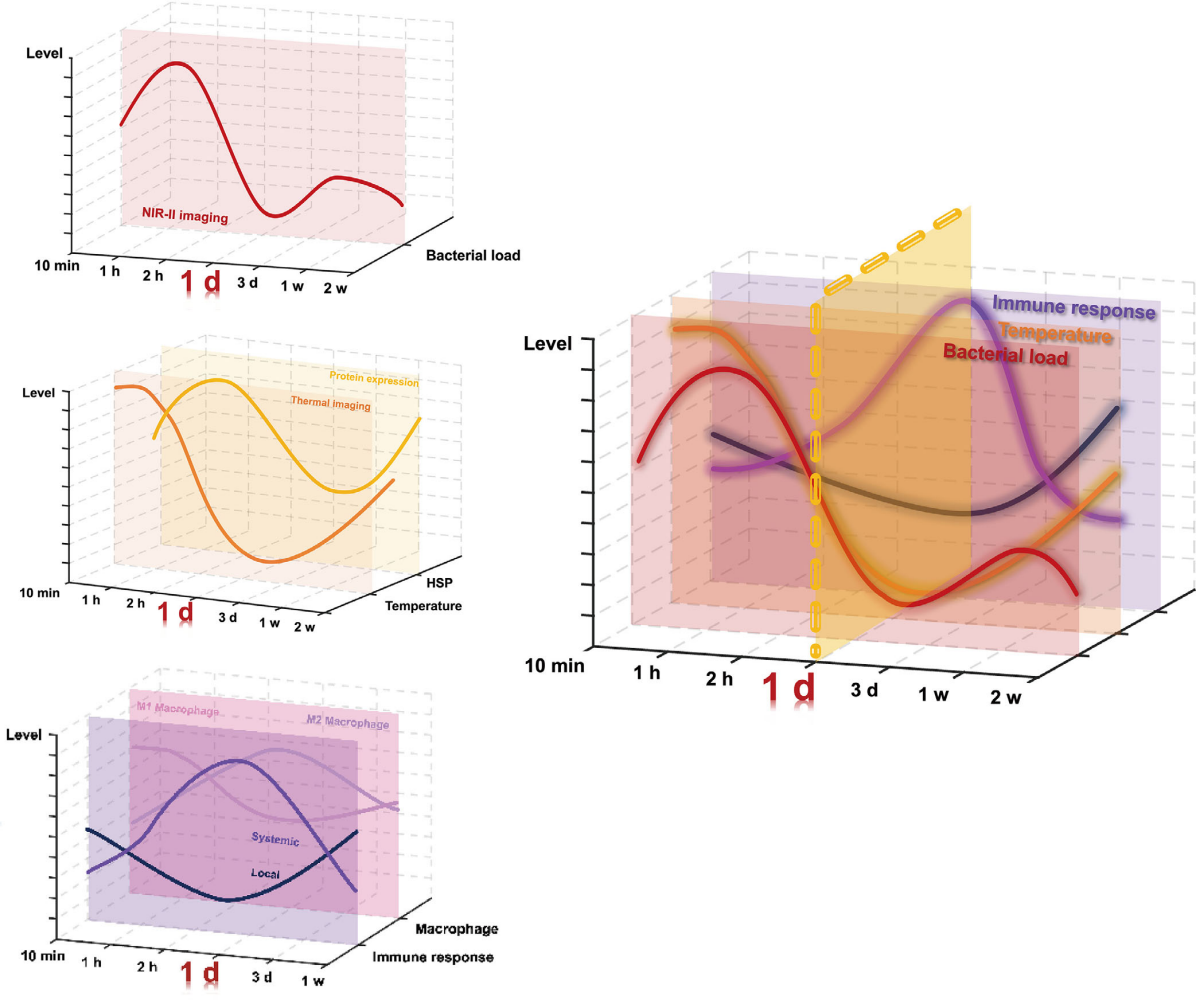
Schematic representation of triadic relation among the bacterial load, temperature and immune response in the joint‐infected mouse model.

The dynamic relationship between the bacterial infection, temperature, and immune response was summarized (Figure 7). In this study, NIR‐II fluorescence imaging and infrared thermography were combined with immune response analysis. It was revealed that 1 day postinfection was the key time point during bacterial infection. Analysis of the triadic relationship and the preliminary inference of the key time points could assist in understanding bacterial infection more accurately and in detail. With the help of a large quantity of real‐time data, the key time point of bacterial infection can be located so that false judgments based on bias due to sampling from various time points can be avoided.

This study demonstrated the real‐time interplay between the bacterial load, temperature, and immune response in vivo based on an infection timeline. As the bacterial load and temperature elevation brought by infection decreased from 10 min to 1 day postinfection, the systemic immune response increased, cause it detected the invasion of the pathogen and began to recruit immune cells. This indicated that before 1 day postinfection, the bacteria underwent a proliferation, which triggered an immediate immune response. In addition, the invasion of bacteria brought with high temperature, as heating and a high fever provide protection against infection by improving the clearance of pathogens from the body.^40^, ^41^ High temperature also induced the increase expression level of HSP60 and HSP90.^40^, ^41^ The increased expression level of both HSPs then played a negative feedback role in regulating body temperature, leading to a decrease in temperature.^11^ Moreover, the delay in the immune response compared with the temperature elevation may be attributed to the fact that immune response are initiated by febrile‐range hyperthermia, which could accelerate pathogen clearance, but also enhance collateral tissue injury.^54^, ^55^, ^56^ After 1 day postinfection, the bacterial load slightly increased and then finally remained at a relatively low level, which was consistent with the general trend of the systemic immune response, whereas the local temperature increased during this period. This indicated that the slight increase in the bacterial load may be assigned to the immune escape of *S. aureus*,^57^ and it didn't activate the high level of immune response again but the infection in the joint still existed. However, the underlying mechanisms remained unclear and required further investigation. In summary, the bacterial load, temperature, and immune response were closely related and determined the progression of bacterial infection.

## 4 CONCLUSIONS

In this study, the prepared NIR‐II fluorescent QDs‐Glu exhibited the ability and stability to label *S. aureus* both in vitro and in vivo, showing enormous potential for in vivo bioimaging. Under the guidance of NIR‐II fluorescence imaging, infrared thermography, and immune response analysis, the triadic relationship between the bacterial load, temperature, and immune response in animal model was elucidated. Furthermore, 1 day postinfection was identified as the key time point during infection. In summary, this study assisted in deciphering the precise course of bacterial infections and provided a reference for rationalizing and optimizing therapeutic regimens during anti‐infection treatment in clinical settings.

## AUTHOR CONTRIBUTIONS

**Huaixuan Sheng:** Conceptualization; data curation; formal analysis; investigation; validation; writing—original draft; writing—review and editing. **Huizhu Li:** Conceptualization; data curation; investigation; validation. **Shunyao Li:** Data curation; formal analysis; software; visualization. **Chengxuan Yu:** Validation. **Yueming Wang:** Resources. **Haichen Hu:** Validation; visualization; writing—review and editing. **Lu Fang:** Software; visualization. **Fuchun Chen:** Data analysis; Resources. **Yanyan Lu:** Data analysis; Resources. **Xiaogang Xu:** Data analysisi; Resources. **Xing Yang:** Validation. **Shiyi Chen:** Project administration; supervision. **Yunxia Li:** project administration; supervision. **Yuefeng Hao:** Manuscript modification; supervision. **Sijia Feng:** Data analysis; project administration; supervision. **Jun Chen:** Conceptualization; Manuscript revise; project administration; supervision.

## CONFLICT OF INTEREST STATEMENT

The authors declare no conflict of interest.

### PEER REVIEW

The peer review history for this article is available at https://www.webofscience.com/api/gateway/wos/peer-review/10.1002/btm2.10656.

## Supporting information


**Figure S1.** NIR‐II fluorescence images of (a1–a6) 1 mL of QDs‐Glu at different exposure times and (a7) graph of PL intensity ratio analyzed from (a1–a6). NIR‐II fluorescence images of QDs‐Glu‐*S. aureus* cocultured for (b) 3 h and (c) 24 h.
**Figure S2.** (a1–a8) NIR‐II fluorescence images of QDs‐Glu injected into knee joint at exposure time of 100 ms with 1300 nm filter. (a9) Graph of PL intensity ratio analyzed from (a1–a8).
**Figure S3.** NIR‐II fluorescence images of QDs‐Glu co‐cultured with (a1–a4) 10^6^ CFU/mL and (b1–b4) 10^8^ CFU/mL of *S. epidermidis* injected into knee joint. (c–e) Graphs of PL intensity ratio analyzed from (a1–a4,b1–b4).
**Figure S4.** NIR‐II fluorescence images of the same mouse infected with QDs‐Glu‐*S. aureus* at exposure time of (a1–a9) 30 ms, (b1–b9) 50 ms, (c1–c9) 100 ms, and (d1–d9) 300 ms. (a10–a11), (b10–b11), (c10–c11), (d10–d11) Graphs of PL intensity ratio analyzed from (a1–a9), (b1–b9), (c1–c9), and (d1–d9). ****p < 0.0001.
**Figure S5.** Graphs of (a) maximum PL intensity ratio and (b) fluorescence area analyzed from NIR‐II fluorescence images infected with *S. aureus*.
**Figure S6.** (a1–a9) NIR‐II fluorescence images of mouse infected with *S. epidermidis*. (a10‐a11) Graphs of PL intensity ratio analyzed from (a1–a9). ****p < 0.0001.
**Figure S7.** (a1–a9) Bright field photographs of blood culture of the same mouse postinfection. (b) Graph of bacterial colonies count analyzed from (a1–a9).
**Figure S8.** Graphs of (a) temperature in three areas, (b) total HSP (HSP60 + HSP70 + HSP90), and (c) HSP70 mRNA expression level postinfection in the joint‐infected mouse model.
**Figure S9.** (a) Bright field photograph and (b) NIR‐II fluorescence image of major organs injected with QDs‐Glu after 2 weeks postinjection with 1300 nm filter. (c–j) Representative H&E staining micrographs of the organ tissues from the mouse injected with QDs‐Glu after 2 weeks postinjection.

## Data Availability

The data supporting this study's findings are available from the corresponding author upon reasonable request.
