# Supplementary material for: Synchronously in vivo real‐time monitoring bacterial load and temperature with evaluating immune response to decipher bacterial infection
Source: Bioeng Transl Med. 2024 Mar 12;9(4):e10656. doi: 10.1002/btm2.10656 (PMC11256147; doi:10.1002/btm2.10656)
Supplement: Supplementary file 1 — Figure S1. NIR‐II fluorescence images of (a1–a6) 1 mL of QDs‐Glu at different exposure times and (a7) graph of PL intensity ratio analyzed from (a1–a6). NIR‐II fluorescence images of QDs‐Glu‐S. aureus cocultured for (b) 3 h and (c) 24 h. Figure S2. (a1–a8) NIR‐II fluorescence images of QDs‐Glu injected into knee joint at exposure time of 100 ms with 1300 nm filter. (a9) Graph of PL intensity ratio analyzed from (a1–a8). Figure S3. NIR‐II fluorescence images of QDs‐Glu co‐cultured with (a1–a4) 106 CFU/mL and (b1–b4) 108 CFU/mL of S. epidermidis injected into knee joint. (c–e) Graphs of PL intensity ratio analyzed from (a1–a4,b1–b4). Figure S4. NIR‐II fluorescence images of the same mouse infected with QDs‐Glu‐S. aureus at exposure time of (a1–a9) 30 ms, (b1–b9) 50 ms, (c1–c9) 100 ms, and (d1–d9) 300 ms. (a10–a11), (b10–b11), (c10–c11), (d10–d11) Graphs of PL intensity ratio analyzed from (a1–a9), (b1–b9), (c1–c9), and (d1–d9). ****p < 0.0001. Figure S5. Graphs of (a) maximum PL intensity ratio and (b) fluorescence area analyzed from NIR‐II fluorescence images infected with S. aureus. Figure S6. (a1–a9) NIR‐II fluorescence images of mouse infected with S. epidermidis. (a10‐a11) Graphs of PL intensity ratio analyzed from (a1–a9). ****p < 0.0001. Figure S7. (a1–a9) Bright field photographs of blood culture of the same mouse postinfection. (b) Graph of bacterial colonies count analyzed from (a1–a9). Figure S8. Graphs of (a) temperature in three areas, (b) total HSP (HSP60 + HSP70 + HSP90), and (c) HSP70 mRNA expression level postinfection in the joint‐infected mouse model. Figure S9. (a) Bright field photograph and (b) NIR‐II fluorescence image of major organs injected with QDs‐Glu after 2 weeks postinjection with 1300 nm filter. (c–j) Representative H&E staining micrographs of the organ tissues from the mouse injected with QDs‐Glu after 2 weeks postinjection. [file BTM2-9-e10656-s001.docx]

**Supporting information**


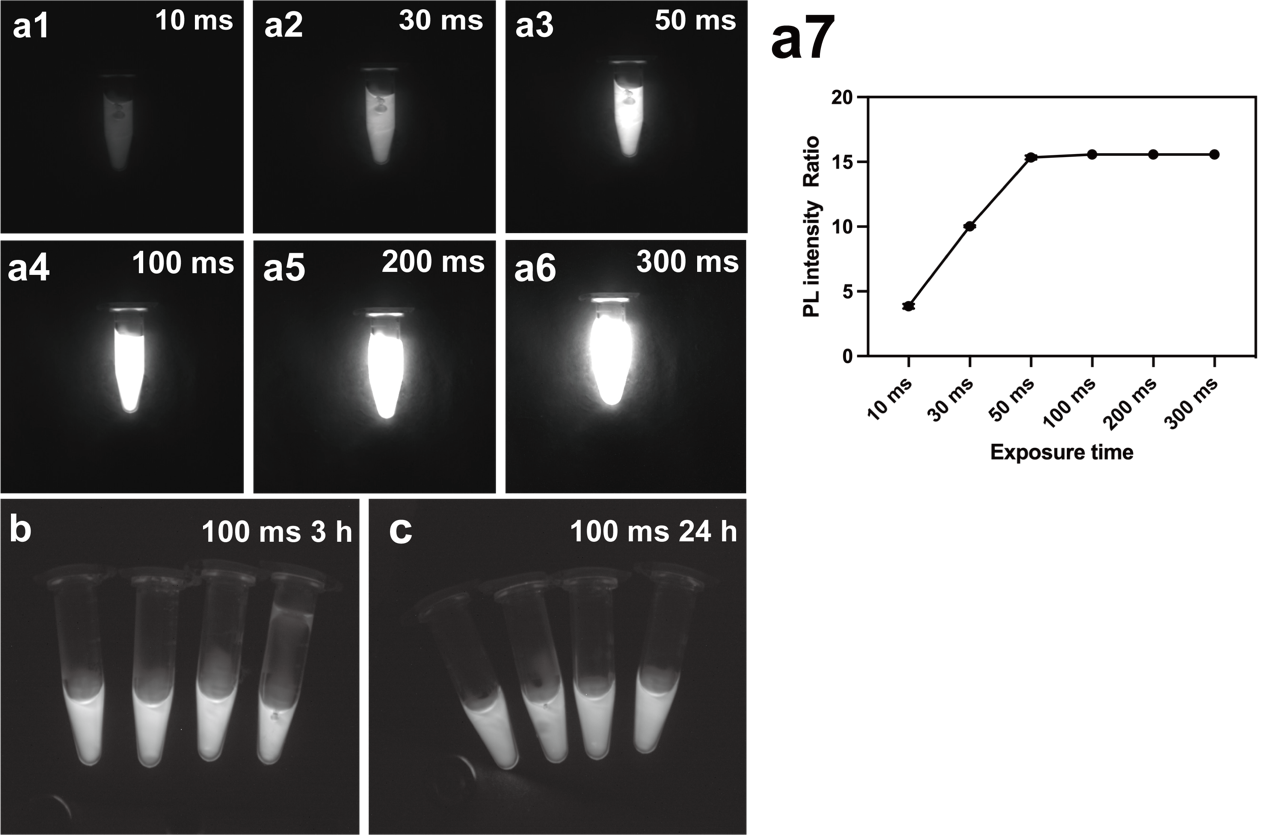


**Figure S1.** NIR-II fluorescence images of a1-a6) 1 mL of QDs-Glu at different exposure times and a7) graph of PL intensity ratio analyzed from a1-a6). NIR-II fluorescence images of QDs-Glu-*S. aureus* co-cultured for b) 3 h and c) 24 h.


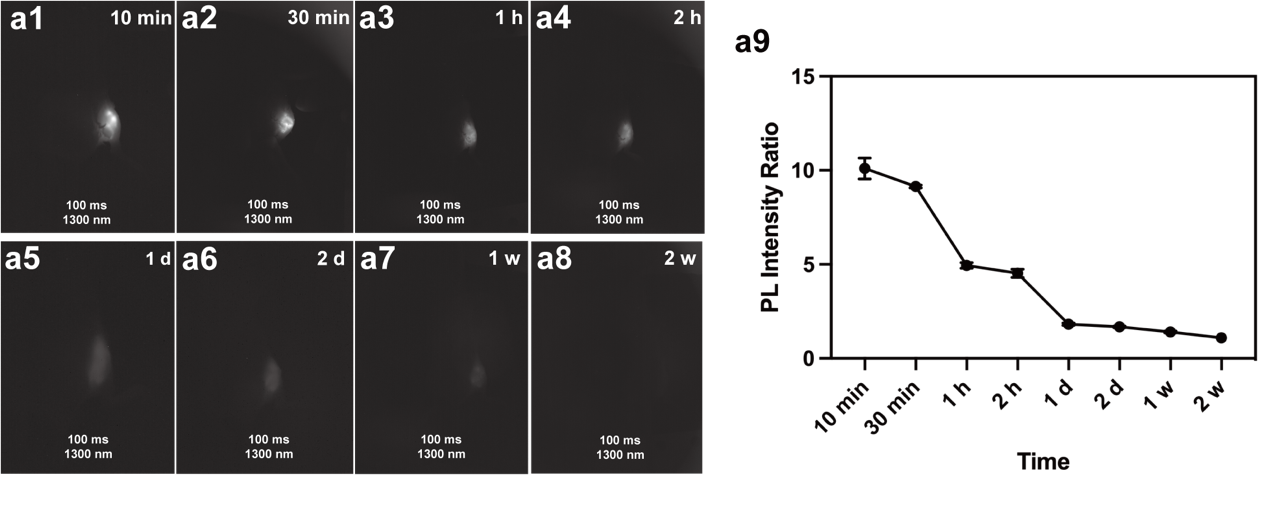


**Figure S2.** a1-a8) NIR-II fluorescence images of QDs-Glu injected into knee joint at exposure time of 100 ms with 1300 nm filter. a9) Graph of PL intensity ratio analyzed from a1-a8).


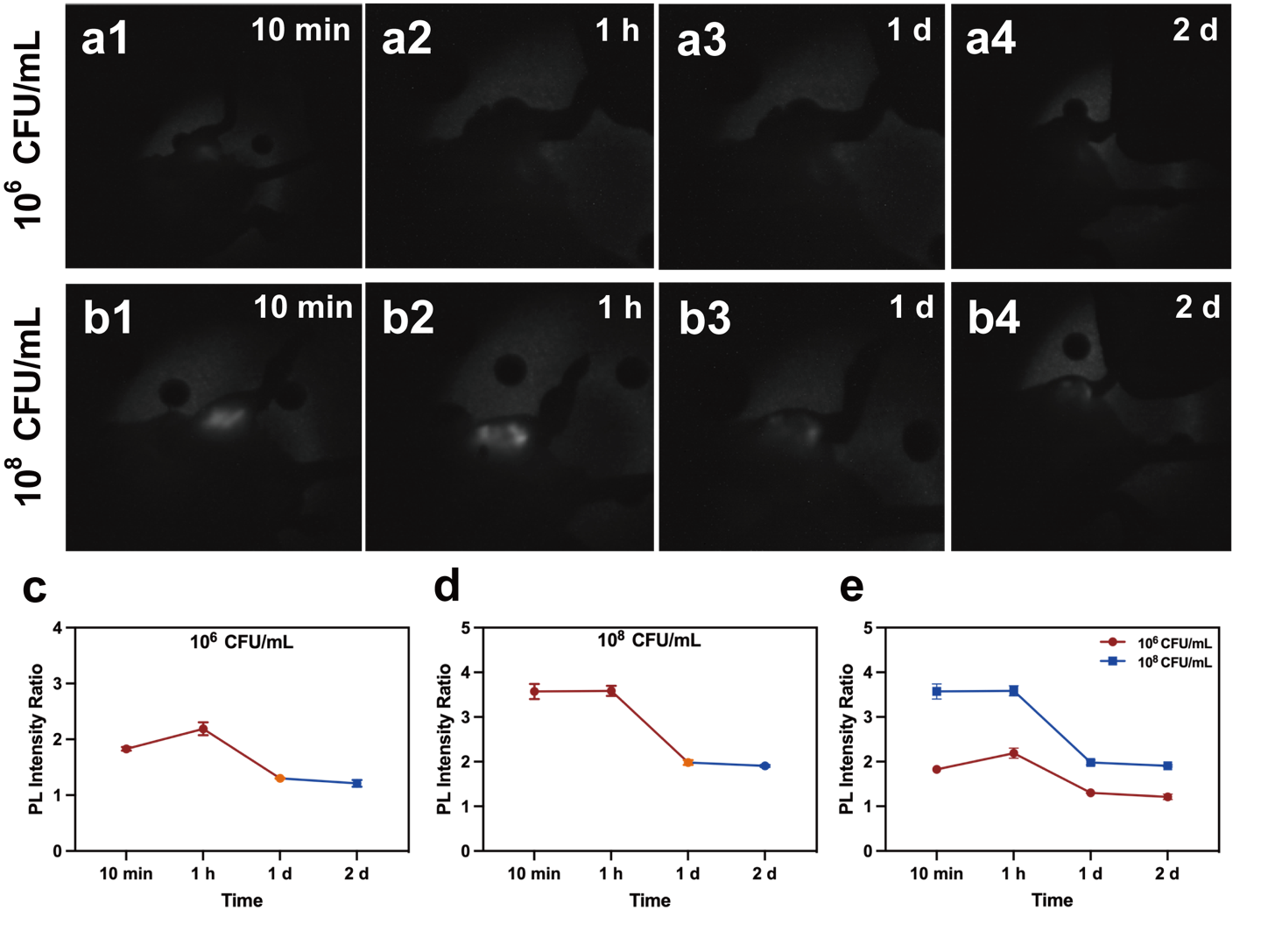


**Figure S3.** NIR-II fluorescence images of QDs-Glu co-cultured with a1-a4) 10^6^ CFU/mL and b1-b4) 10^8^ CFU/mL of *S. epidermidis* injected into knee joint. c-e) Graphs of PL intensity ratio analyzed from a1-a4), b1-b4).


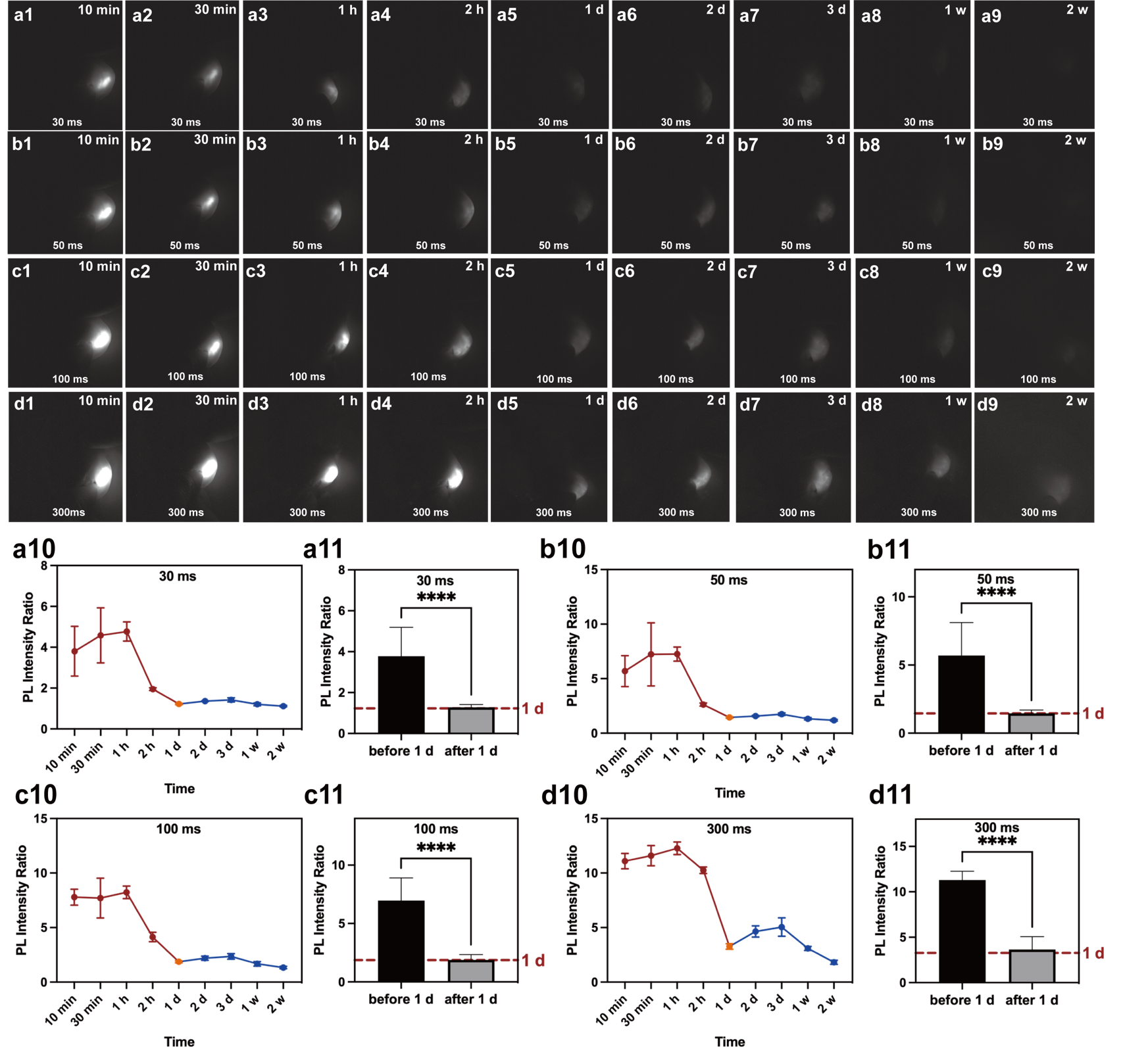


**Figure S4.** NIR-II fluorescence images of the same mouse infected with QDs-Glu-*S. aureus* at exposure time of a1-a9) 30 ms, b1-b9) 50 ms, c1-c9) 100 ms and d1-d9) 300 ms. a10-a11, b10-b11, c10-c11, d10-d11) Graphs of PL intensity ratio analyzed from a1-a9), b1-b9), c1-c9), d1-d9). ****; *p* < 0.0001.


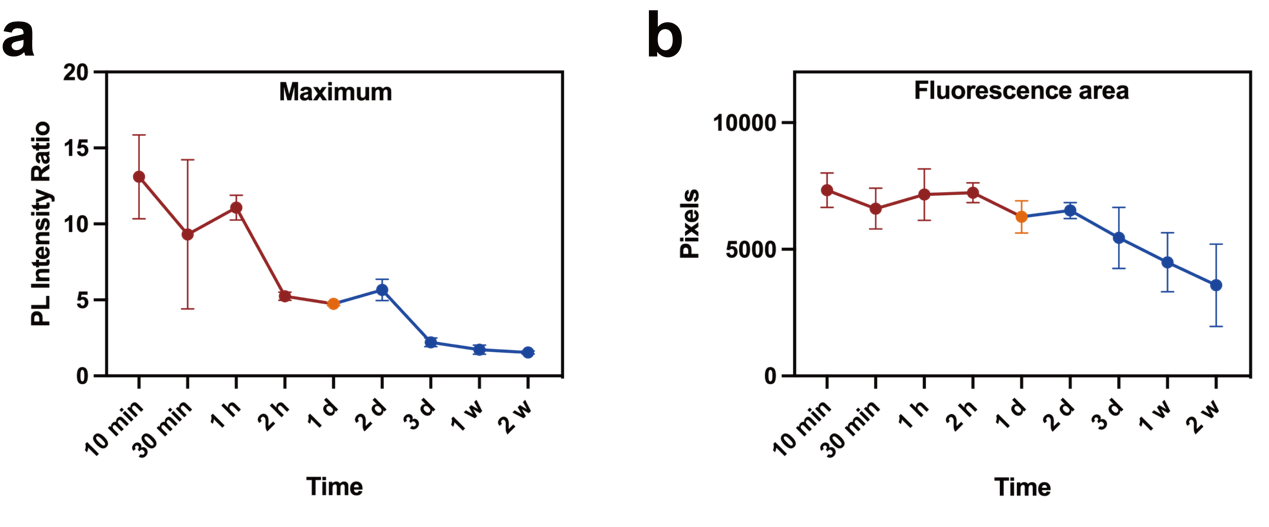


**Figure S5.** Graphs of a) maximum PL intensity ratio and b) fluorescence area analyzed from NIR-II fluorescence images infected with *S. aureus*.


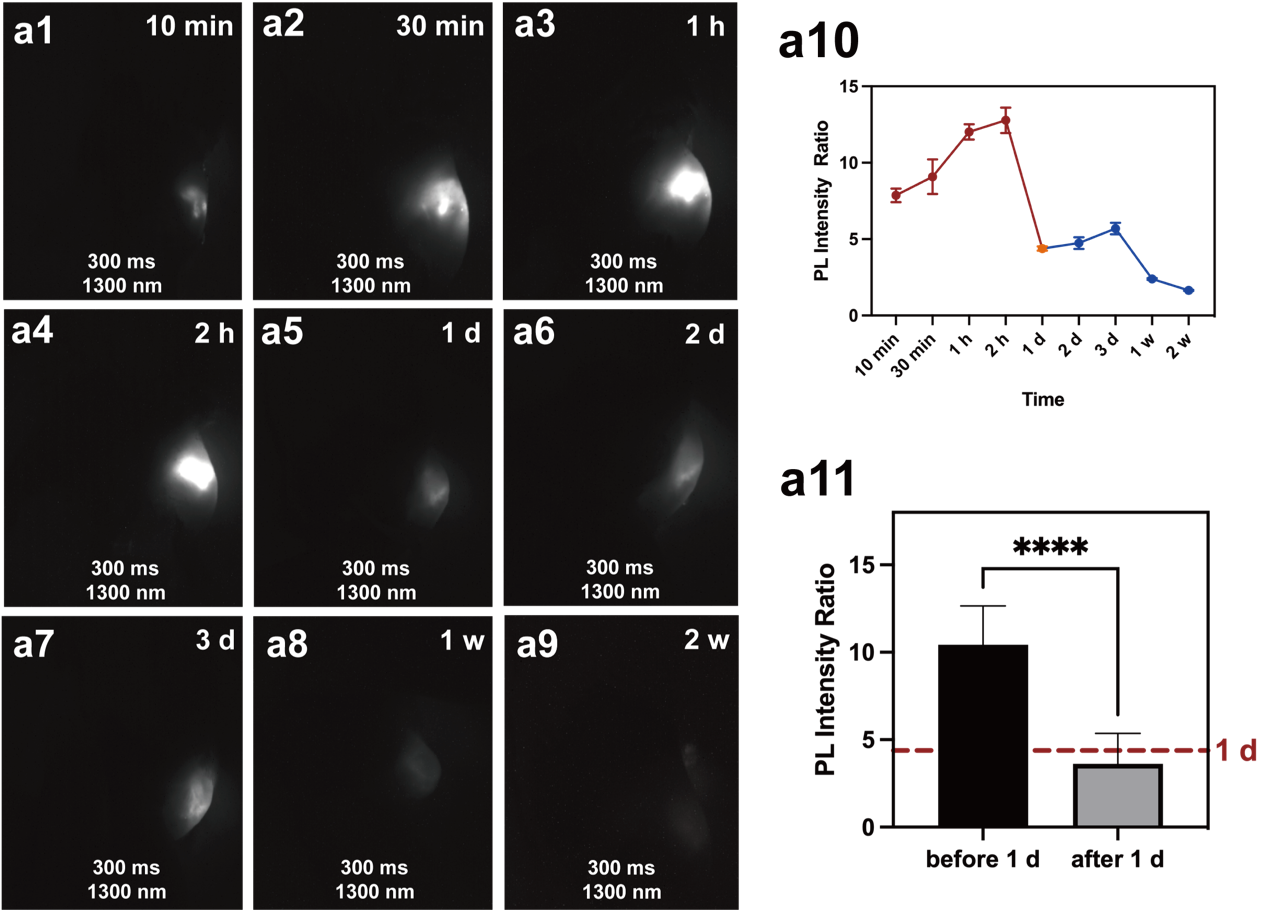


**Figure S6.** a1-a9) NIR-II fluorescence images of mouse infected with *S. epidermidis*. a10-a11) Graphs of PL intensity ratio analyzed from a1-a9). ****; *p* < 0.0001.


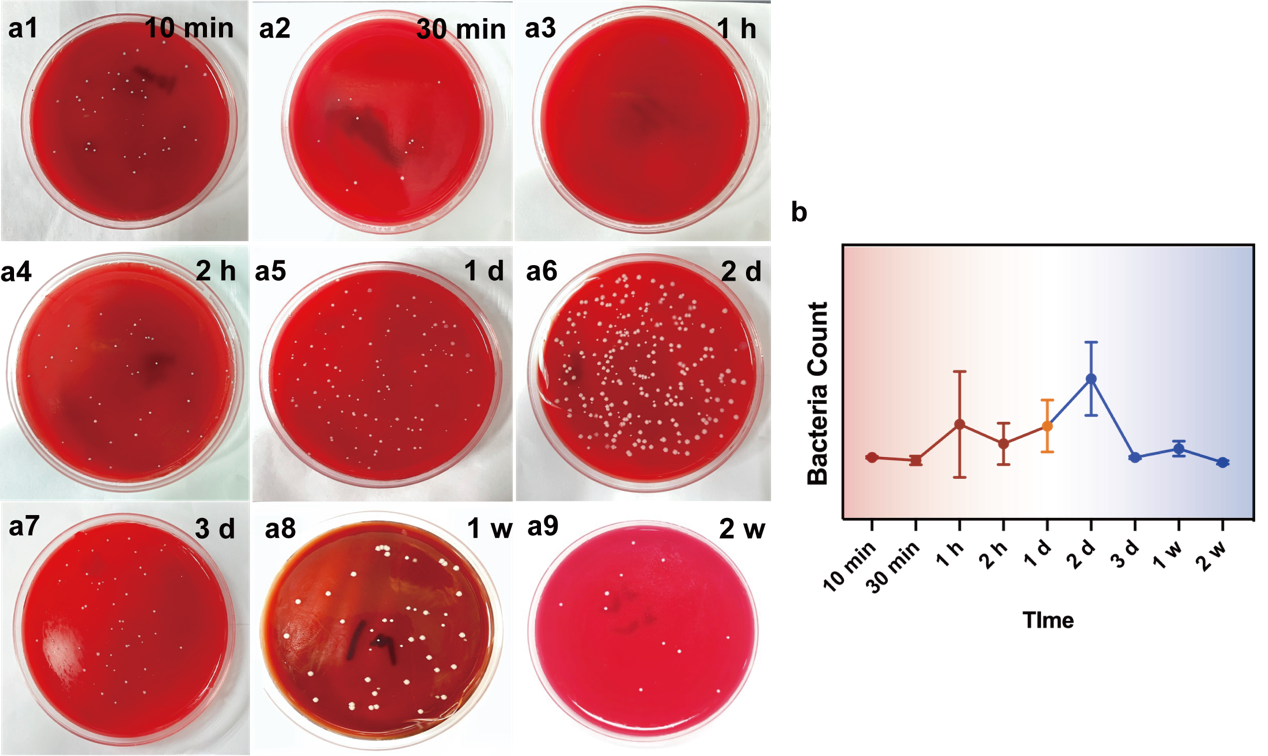


**Figure. S7.** a1-a9) Bright field photographs of blood culture of the same mouse postinfection. b) Graph of bacterial colonies count analyzed from a1-a9).


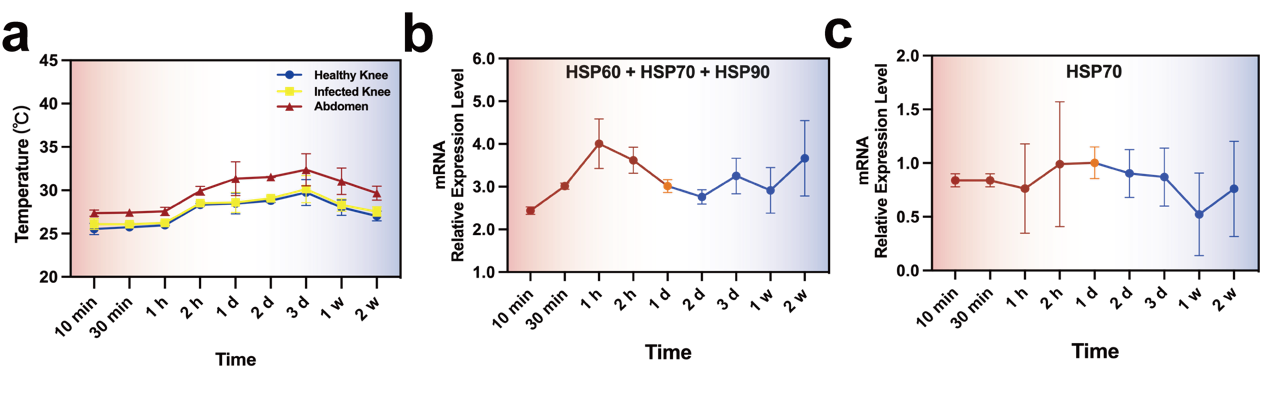


**Figure S8.** Graphs of a) temperature in three areas, b) total HSP (HSP60 + HSP70 + HSP90) and c) HSP70 mRNA expression level postinfection in the joint-infected mouse model.


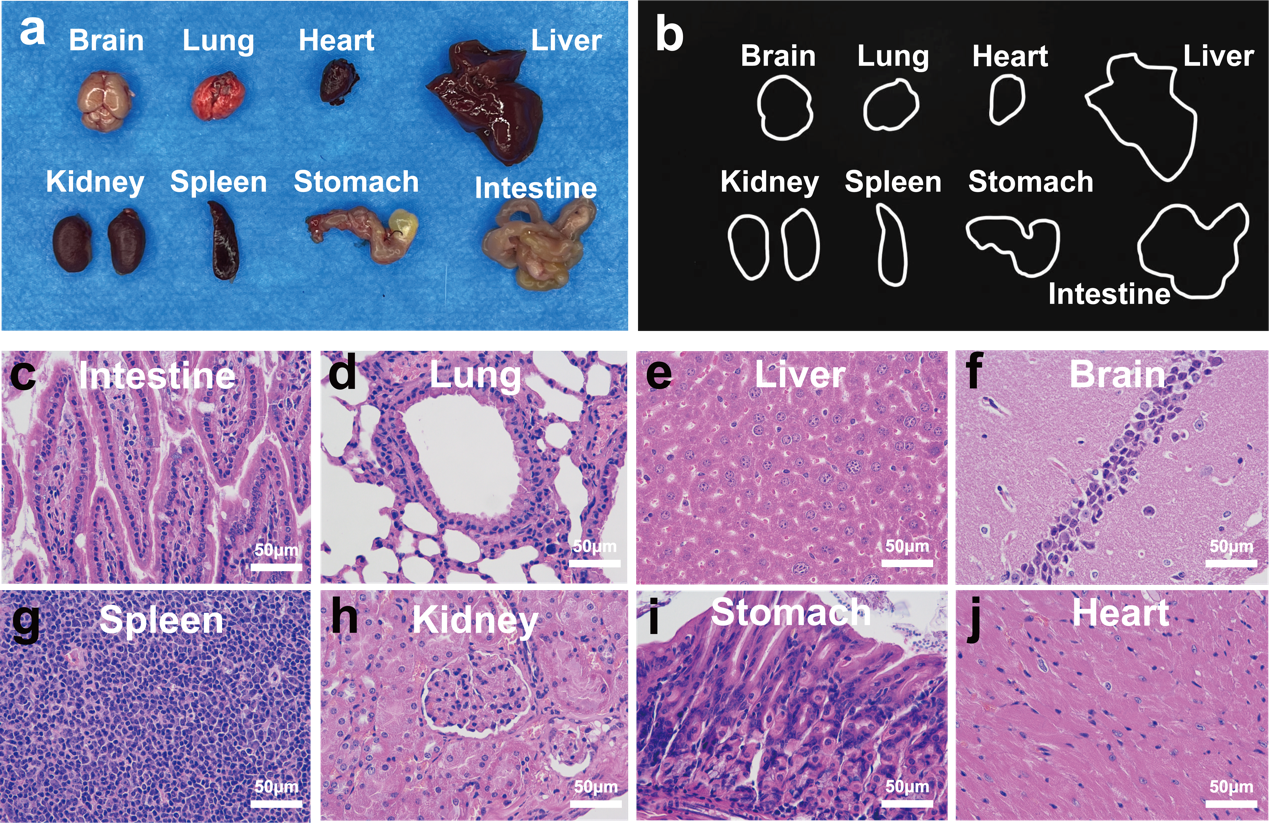


**Figure S9.** a) Bright field photograph and b) NIR-II fluorescence image of major organs injected with QDs-Glu after 2 w post-injection with 1300 nm filter. c-j) Representative H&E staining micrographs of the organ tissues from the mouse injected with QDs-Glu after 2 w post-injection.
